# Meticulous Closure of Mesenteric Defects Effectively Reduces the Incidence of Internal Hernia After Laparoscopic Roux-en-Y Gastric Bypass

**DOI:** 10.1007/s11695-024-07306-1

**Published:** 2024-06-20

**Authors:** Aurélie Vuagniaux, Beatriz Barberá-Carbonell, Anna Dayer, Styliani Mantziari, Michel Suter

**Affiliations:** 1 Department of Visceral Surgery, University Hospital (CHUV), Lausanne, Switzerland; 2 Department of Surgery, Riviera-Chablais Hospital, Centre Médico-Chirurgical de L’Obésité Riviera-Chablais, Rte Des Tilles 6A, 1847 Rennaz, Switzerland; 3 Faculty of Biology and Medicine, University of Lausanne, Lausanne, Switzerland

**Keywords:** Gastric bypass, Complication, Internal hernia

## Abstract

**Introduction:**

Internal hernia (IH) after Roux-Y gastric bypass (RYGB) can lead to extended small bowel ischemia if it not recognized and treated promptly. The aim of this study is to show whether improvement in mesenteric defect (MD) closure reduces the incidence of IH.

**Patients and Methods:**

Retrospective analysis of prospectively collected data from our database including all patients who underwent laparoscopic RYGB between 1999 and 2015. The usual technique was a retrocolic/retrogastric RYGB. We divided patients in four groups according to the closure technique for MD and compared incidences of IH between groups. All patients had at least 8 years of follow-up.

**Results:**

A total of 1927 patients (1497 females/460 males, mean age of 41.5 ± 11 years) were operated. A retrocolic/retrogastric RYGB was performed in 1747 (90.7%) and an antecolic RYGB in 180 patients. Mean duration of follow-up was 15 (8–24) years. 111 patients (5.8%) developed IH, the majority through the jejunojejunostomy (JJ, 3.7%) and Petersen (1.7%) defects. With improvement of closure technique, the incidence decreased over time, from 12.9% in the group with separate sutures to 1.05% in the most recent group with running non-absorbable sutures and an additional purse-string at the JJ defect (*p* < 0.0001).

**Conclusion:**

Meticulous closure of MD during RYGB is a very important step that significantly reduces the IH risk after RYGB, even with a retrocolic/retrogastric anatomy. Using running non absorbable braided sutures and an additional purse-string suture at the JJ is the most effective technique, but a small IH risk persists. A high index of suspicion remains necessary in patients who present with acute abdominal pain after RYGB.

**Graphical Abstract:**

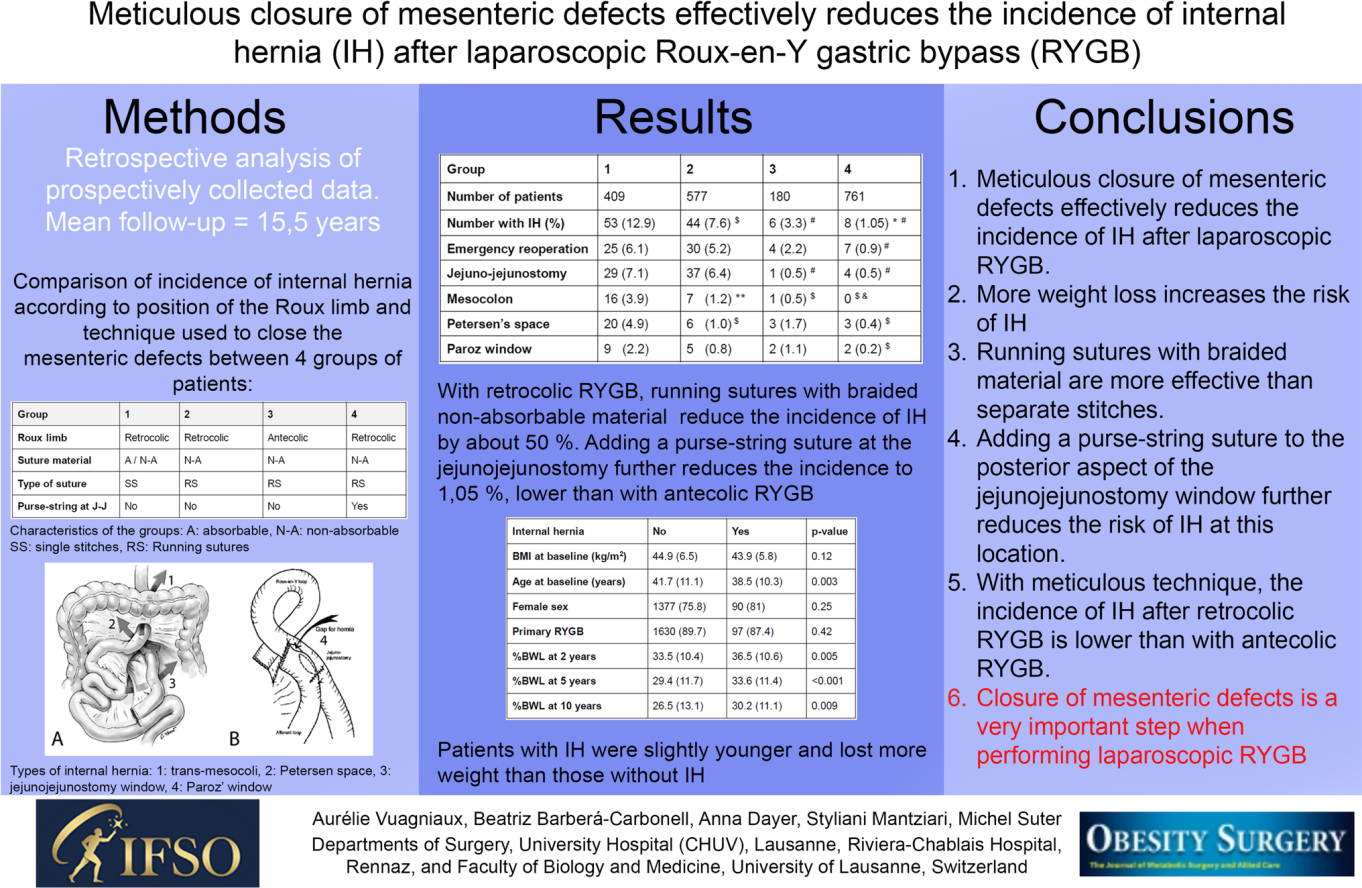

## Introduction

Roux-en-Y gastric bypass (RYGB) is currently the second most performed metabolic/bariatric surgical procedure worldwide. With prevalence varying between countries and surgeons, it is still the most common operation in Switzerland. Despite good long-term outcomes regarding weight loss, improvement of obesity-related complications, and quality of life [1–4], RYGB has recently been outnumbered by sleeve gastrectomy (SG), mainly because the latter is perceived as easier to perform and associated with reduced early morbidity/mortality, also avoiding the risk of late internal hernia (IH).

IH is a feared complication that can develop due to adhesions or a mesenteric gap after several abdominal surgical procedures and is one of the leading causes of abdominal pain [5] and small bowel obstruction (SBO) after RYGB [6]. After RYGB, IH is often caused by incarceration of small bowel through a mesenteric defect (MD) created during the index operation. While IH was relatively unusual after open RYGB, with rates between 1 and 4.7% [7, 8], its incidence increased after introduction of laparoscopic RYGB, with reported rates as high as 14,4% [9], notably because laparoscopy results in fewer adhesions than open surgery but also because closure of mesenteric defects is more technically challenging. This problem was quickly recognized during the early years of laparoscopy. Consequently, several authors already suggested to close the MD more than 20 years ago [10–12]. Others believed that non-closure of the MD was not the main cause of IH and that other factors (position of the Roux-limb, division of the greater omentum) were more important [13–15]. The debate lasted for years, albeit with a growing number of authors supporting systematic closure [16–20]. A first randomized controlled trial (RCT) involving 105 patients concluded in 2015 that closing the MD did not influence the risk of IH, but this study was largely underpowered [21]. In 2016, a large multicenter RCT from Scandinavia involving 2507 patients, comparing non-closure with closure using running non-absorbable braided sutures in patients undergoing an antecolic RYGB, concluded that the risk of reoperation for SBO was decreased in the closure group, although the risk of early post-operative kinking at the jejunojejunostomy was increased [22]. More recently, four systematic reviews and meta-analysis also concluded that closure of MD reduces the risk of IH, SBO and reoperation after RYGB [23–26]. The long-term results of the Scandinavian RCT showed that, despite closure, the cumulative 10-year incidence of IH remained non-neglectable at a 7.8% rate compared with 14.9% in the non-closure group [27]. Despite the large body of evidence showing that systematic closure of MD should be the rule during RYGB surgery, a recent online survey among 34 countries revealed that 16/136 (11.7%) responding surgeons never closed any defect [28].

While closing MD has now clearly been established as a mandatory step at completion of RYGB, the precise technique for closure is still debated. The use of a mesh to reinforce closure has even been described [29, 30]. As others, and despite systematic closure of all MD as of the beginning of our experience with laparoscopic RYGB, we have observed a high incidence of IH initially (12.9%), which led us to adjust the closing technique to improve permanent closure and reduce the risk of IH. The aim of the present paper is to show that the implementation of a meticulous closing technique for MD can markedly decrease the incidence of IH and their associated risks in RYGB patients.

## Patients and Methods

All patients who underwent primary or revisional laparoscopic RYGB for obesity between 1999 and 2015 in our two institutions, led by the same bariatric surgeon, were included in this retrospective study. Our prospectively maintained common bariatric database was analyzed regarding the occurrence of IH in relationship with the precise operative technique used to close the MD.

Indications for RYGB were a body mass index (BMI) ≥ 40 kg/m [2], or a BMI ≥ 35 kg/m [2] with obesity-related medical conditions until 2010, and BMI ≥ 35 kg/m^2^ as of 2011, after failure of conservative treatment and evaluation by a multidisciplinary team (endocrinologist, psychiatrist/psychologist, dietitian, and bariatric surgeon).

Patients were operated by laparoscopy with a standardized technique by the same surgical team in both institutions. The standard technique involved creation of a small 10–20 ml gastric pouch along the upper portion of the lesser curvature using a linear stapler, division of the jejunum about 50 cm from the angle of Treitz and creation of a retro-colic and retro-gastric Roux-en-Y limb with a circular-stapled gastrojejunostomy (GJS) using a 21-mm stapler. A latero-lateral jejunojejunostomy between the biliary limb and the Roux limb was performed 100 or 150 cm distal from the GJS using a linear stapler depending on whether the patient’s BMI was below or above 50. As of 2009, one surgeon started to use an antecolic and ante-gastric Roux limb. Most RYGB, however, were performed with a retro-colic and retro-gastric alimentary limb.

**Fig. 1:**
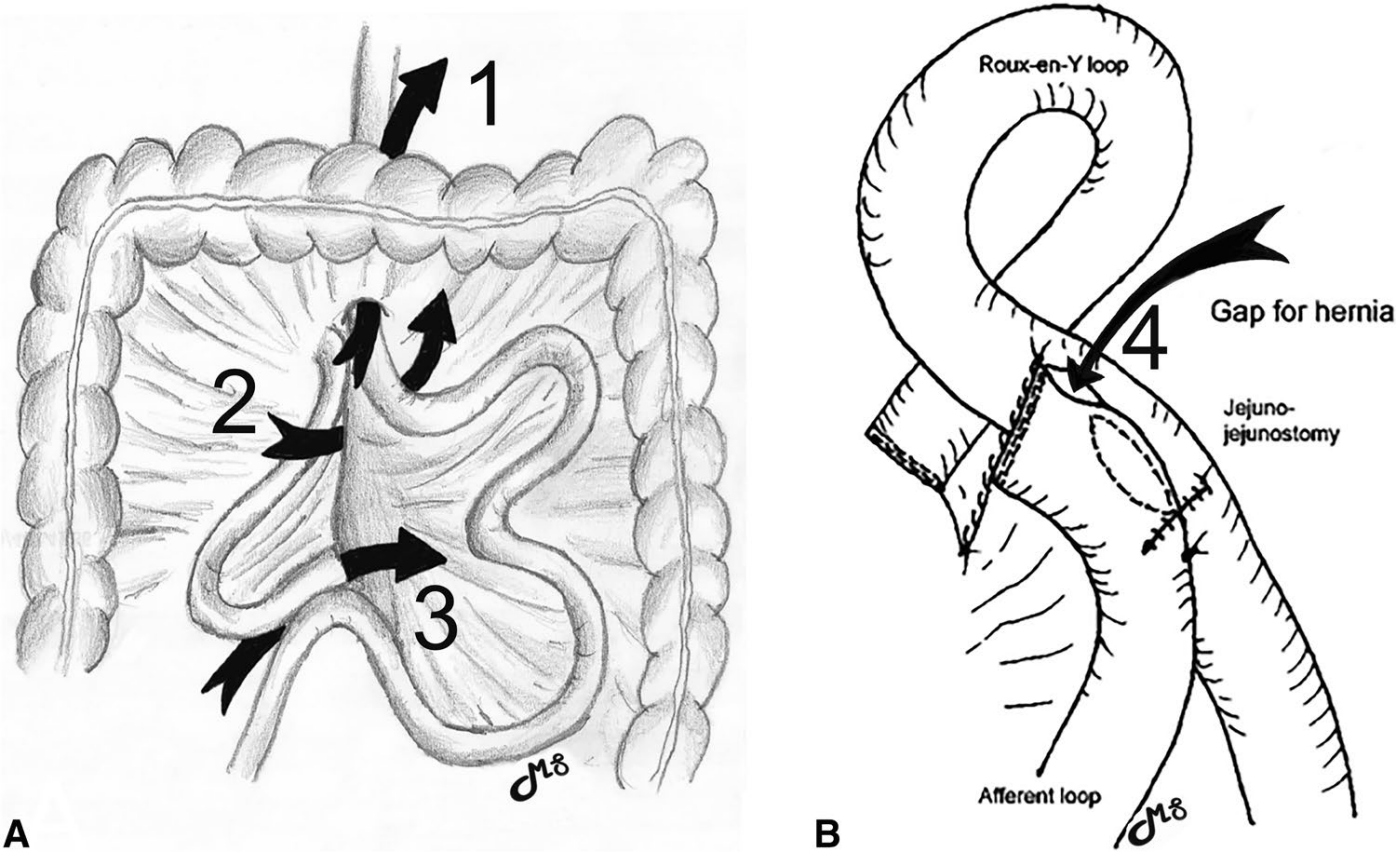
**A** and (**B**) Types and positions of IH after retrocolic-retrogastric RYGB. 1, transmesocolic window; 2, Petersen’s space; 3, jejunojejunostomy window; 4. Paroz’ window

Except for two patients in our early experience where exposure was too limited, the mesenteric defects were closed at the end of the procedure in all patients. At the beginning of our experience, we closed the classical three defects (Fig. 1A: Petersen window, mesocolic window (MC), and jejunojejunostomy (JJ) window). After observing the first case of IH though the space between the JJ and the anti-obstruction stitch in 2007 (Fig. 1B), we also routinely closed this latter defect using a figure-of-eight or a short running non-absorbable sutures [31]. Initially, closure was done using separate absorbable Vicryl® sutures. As we observed a relatively large number of IH with absorbable sutures, we switched to non-absorbable separate stitches in 2004. With growing experience, we became stricter in the closure technique of the MD and introduced further changes. Separate stitches were replaced by running non-absorbable sutures using a braided suture material (Ethibond®). Later, realizing that most of our internal hernias were through the defect at the JJ, we added a purse-string suture on its posterior aspect for better closure. The technique has remained identical since then. All patients who had an antecolic Roux limb were operated on by the same surgeon who had extended experience with the retro-colic technique and MD closure, and all had their defects closed (Petersen and JJ) with running non-absorbable sutures (Ethibond®).

We divided patients into four groups according to the position of the Roux limb and the technique of closure (Table 1). Internal hernia was defined as an open mesenteric window visualized during subsequent surgery, related or not to RYGB, with or without associated clinical symptoms, and with or without bowel herniation through the defect at the time of revision.

**Table 1:** Characteristics of the four groups (*A*, absorbable; *N-A*, non-absorbable; *J-J*, jejunojejunostomy; *SS*, separate stitches; *RS*, runnings sutures)

Group	1	2	3	4
Roux limb	Retrocolic	Retrocolic	Antecolic	Retrocolic
Suture material	A / N-A	N-A	N-A	N-A
Type of suture	SS	RS	RS	RS
Purse-string at J-J	No	No	No	Yes

The primary endpoint of the study was the incidence of IH in each group. Demographic data, location of mesenteric window, and clinical presentation of internal hernias were evaluated as secondary endpoints. Although IH can develop any time after RYGB, most hernia present during the first post-operative years according to our experience and that of other authors [11, 18, 32] In order to have a duration of follow-up of at least eight years for all the patients, only those operated until 2015 were analyzed.

All patients were included in the COOL cohort (COhorte Obésité Lausanne) and all provided written informed consent for deidentified use of their data for scientific purpose. The use of data from this cohort was approved by the local Ethics committee (CER-VD 304/15).

Categorical variables were expressed as numbers (%) and compared with the Chi-squared test or the Fisher’s exact test. Continuous variables were expressed as mean ± standard deviation (SD) and compared using the *t*-test. A two-sided *p*-value < 0.05 was considered threshold for statistical significance. All analysis were performed using the STATA/SE 17.0 software for windows (StataCorp LLC, College Station TX, USA).

## Results

A total of 1927 patients underwent laparoscopic RYGB and were prospectively included in the database between 1999 and 2015. RYGB was a primary operation in 1778 patients but was done as a secondary procedure for complication(s) and/or recurrence of weight after vertical banded gastroplasty or gastric banding in 249 patients. Table 2 shows the patients’ demographic data at baseline.

**Table 2:** Demographic and anthropometric characteristics of the patients at baseline (#: *p* < 000.1 versus 1 and 2, ##*p* < 0.0001 versus 3). *BMI*, body mass index; *SD*, standard deviation

Group	1	2	3	4	*p*-value
Number	409	577	180	761	N/A
Females (%)	312 /76.3)	442 (76.6)	152 (84.4)	561 (73.3)	0.02
Mean age (SD)	39.3 (10.2)	40.6 (10.7)	41.5 (10.4)	43.3 (11.6) ^#^	< 0.001
Mean BMI (SD)	45.4 (7.5)	44.7 (5.7)	43.9 (5.2)	44.8 (6.7)	0.08
Primary procedures (%)	367 (89.7)	529 (91.7)	174 (96.7)	657 (86.3) ^##^	< 0.001

More than 90% of RYGB were performed with a retro-colic and retro-gastric alimentary limb, whereas in 180 (9.3%) patients, an ante-colic technique was used because of surgeon’s preference. Patient age increased slightly along our growing experience, and so did the proportion of male patients. Duration of follow-up is at least 8 years for all patients, with a mean of 15 (range 8–24) years and a median of 15.5 years. The follow-up rate of the entire cohort is 90.6%, 74.4%, and 59% after 10, 15, and 20 years respectively.

**Fig. 2:**
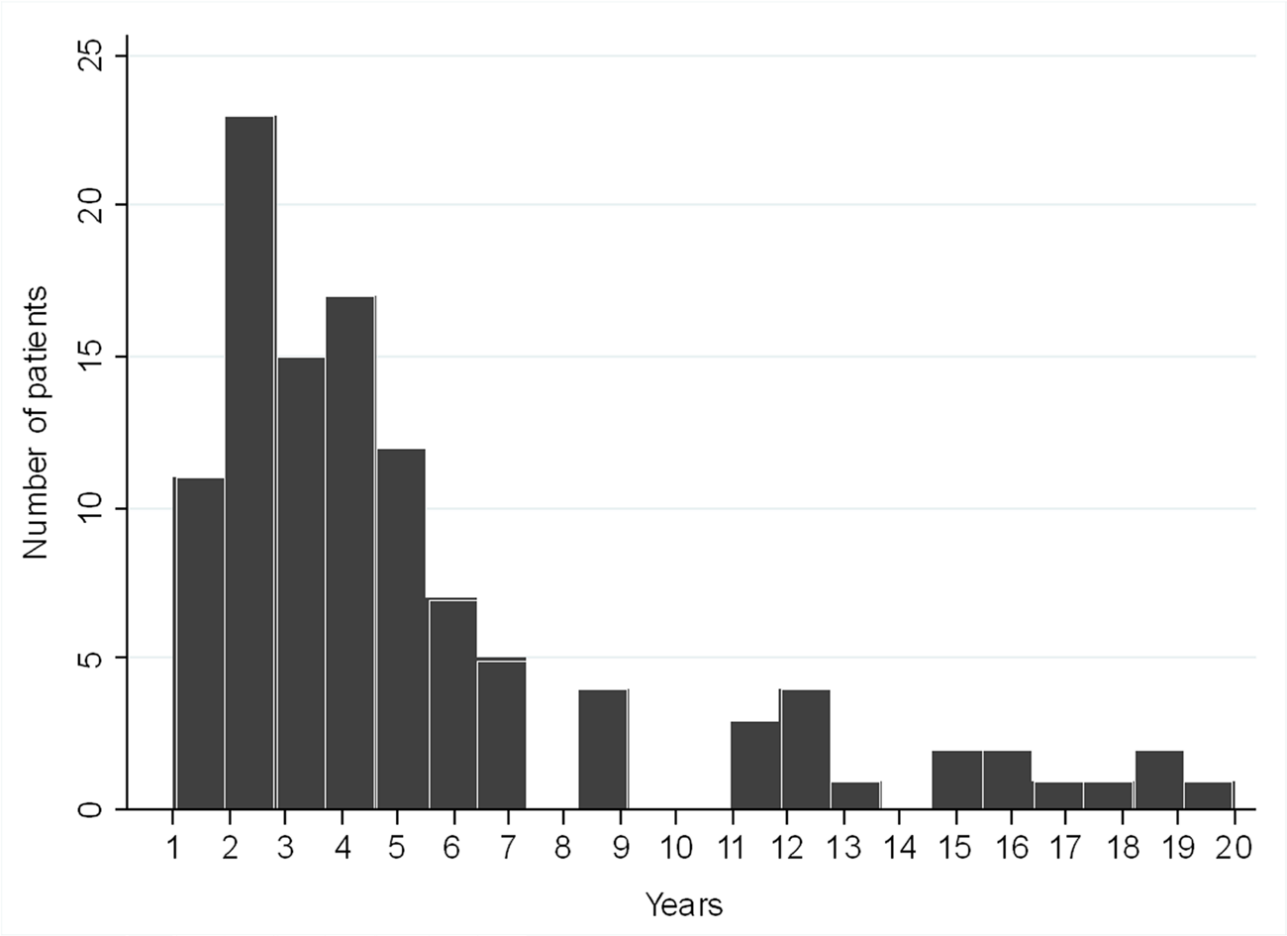
Delay between surgery and IH presentation

A total of 111 (5.8%) patients developed an IH during follow-up. Of these, two of our early patients in whom the MD were not closed during RYGB because of technical difficulty developed IH as an early complication 7 and 15 days after surgery, requiring reoperation. All other patients presented with IH beyond the first post-operative month. There was no difference in the incidence of IH between primary and reoperative RYGB. Figure 2 shows the delay between initial surgery and IH presentation. 90 (80%) patients developed an IH during the first 7 years after RYGB. Table 3 compares some baseline characteristics and weight loss data expressed as %total body weight loss (%TWL) between patients with and without IH. There were no differences between groups in patients’ characteristics at baseline except for younger age in patients with IH. Patients with IH, however, presented significantly higher %TWL at all time intervals (2, 5, and 10 years after RYGB) than those without IH.

**Table 3:** Comparison of baseline and weight loss data in patients with and without internal hernia. Data are expressed as mean (SD) for numerical values and number (%) for categorical values. *BMI*, body mass index; *RYGB*, Roux-en-Y gastric bypass; *%TWL*, % total body weight loss

Internal hernia	No	Yes	*p*-value
BMI at baseline (kg/m [2])	44.9 (6.5)	43.9 (5.8)	0.12
Age at baseline (years)	41.7 (11.1)	38.5 (10.3)	0.003
Female sex	1377 (75.8)	90 (81)	0.25
Primary RYGB	1630 (89.7)	97 (87.4)	0.42
%TWL at 2 years	33.5 (10.4)	36.5 (10.6)	0.005
%TWL at 5 years	29.4 (11.7)	33.6 (11.4)	< 0.001
%TWL at 10 years	26.5 (13.1)	30.2 (11.1)	0.009

Table 4 shows the number of patients with an IH in the different groups and their location. Since several patients had more than one open MD at revision, the total number of IH is superior to the number of affected patients. In patients with a retro-colic and retro-gastric Roux limb, the prevalence of IH decreased by one-third when moving from separate stitches to running sutures. The addition of a purse-string suture at the JJ markedly contributed to a further reduction with a 1.05% rate in patients operated from 2008 to 2015, significantly lower than the two other retro-colic groups. This was even significantly lower than the 3.3% rate observed in patients with an antecolic Roux limb.

**Table 4:** Number of patients with IH (internal hernia), emergency operations and the different locations of IH in the different groups. ($*p* ≤ 0.01 versus 1, #*p* < 0.001 versus 1 and 2, *p = 0.03 versus 3, **p = 0.03 versus 1, &*p* = 0.002 versus 2)

Group	1	2	3	4
Number of patients	409	577	180	761
Number with IH (%)	53 (12.9)	44 (7.6) ^$^	6 (3.3) ^#^	8 (1.05) * ^#^
Emergency reoperation	25 (6.1)	30 (5.2)	4 (2.2)	7 (0.9) ^#^
Jejuno-jejunostomy	29 (7.1)	37 (6.4)	1 (0.5) ^#^	4 (0.5) ^#^
Mesocolon	16 (3.9)	7 (1.2) **	1 (0.5) ^$^	0 ^$ &^
Petersen’s space	20 (4.9)	6 (1.0) ^$^	3 (1.7)	3 (0.4) ^$^
Paroz window	9 (2.2)	5 (0.8)	2 (1.1)	2 (0.2) ^$^

Emergency surgery was performed for IH repair in 66 (59.4%) patients and a small bowel resection was required in three of them. The other 44 (39.6%) patients were reoperated on electively, and this information is missing for one patient. Of patients reoperated on electively, 7 were reoperated for another reason than IH, but had an IH diagnosed and repaired during this procedure. A laparoscopic approach was used in 102 (91.9%) patients, of which 14 required conversion to open surgery, and 9 patients had an open approach.

## Discussion

This study shows that the implementation of a meticulous technique for closure of MD effectively reduces the incidence of IH after RYGB. In the most recent group of patients with a retrocolic and retrogastric Roux limb, the IH rate after a mean of 11.5 (8–15) years was 1.05%. The most common sites for IH were the JJ window and the Petersen defect, even in the most recent group. IH through the mesocolic defect, initially observed during our early experience, completely disappeared in the last group of patients. This 1.05% rate compares favorably with the long-term results reported recently from the Scandinavian RCT where the 10-year cumulative IH rate was 7.8% [27] or with the rates reported after closure of MD in four recent meta-analysis comparing the incidence of IH with or without systematic closure of MD [23–26]. In the present series, TWL was higher in patients who developed IH. Schneider et al. also reported lower BMIs in patients with IH than in the rest of their cohort [33]. With more weight loss, the mesenteries become thinner, which probably facilitates herniation through a small defect.

IH is a feared complication after RYGB that can lead to extended bowel necrosis requiring extensive resection resulting in short-bowel syndrome if not recognized and treated promptly, so that its prevention is an essential part of the procedure. While relatively high rates were reported in the early days, many authors individually realized that the systematic closure of MD reduces the incidence of IH and SBO and modified their technique accordingly [34–39]. One large multicenter RCT and four meta-analysis came to the same conclusions [22–27]. Some complications related to closure (bleeding or hematoma in the mesentery) or possibly related to closure (kink at the JJ) have been reported. Closure has also been associated with an increased risk of SBO due to adhesions [39], but a recent large study involving more than 5000 patients showed that systematic closure is safe if the appropriate technique is used [40]. Despite some ongoing debate, we can safely confirm that, based on the current evidence from the literature and our own experience, routine closure of all MD is a very important step in laparoscopic and probably also robot-assisted RYGB.

Most surgeons perform RYGB with the Roux limb placed in an antecolic and antegastric position. This technique is often perceived as easier and many surgeons consider that having only two mesenteric windows, as opposed to three when a retrocolic route is used, should reduce the risk of IH [12, 14, 20]. In 2016, a meta-analysis comparing the antecolic with the retrocolic route concluded that the former reduces the risk of IH [41]. The authors of another meta-analysis published the same year found reduced rates of both IH and SBO with the antecolic technique but felt that a RCT was necessary to draw definitive conclusions [42]. Carmody et al. and Miyashiro et al. both reported low rates of IH after retrocolic RYGB [17, 19]. In the present study, a very low rate of IH has been achieved with meticulous closure of all MD in patients operated with a retrocolic Roux limb. Furthermore, the majority of IH developed at the JJ and Petersen defect with only a minority of IH at the MC defect in our early experience, and none in the most recent group. We conclude that, provided the MC defect is closed appropriately, the risk of IH through the MC window is very low after retrocolic RYGB.

The technique used to close MD has been a matter of debate for a long time, and several methods have proved to reduce the incidence of IH compared with non-closure. Some surgeons use separate stitches and others use metal clips or staples, both resulting in separate sutures, but most use running sutures. With separate sutures, there is a theoretical increased risk that, with thinning of the mesentery resulting from weight loss, small gaps between clips or sutures increase in size over time and facilitate the development of IH. This risk is probably lower with running sutures provided the bites are not too far apart. In the past, even the use of mesh has been suggested to increase adhesions at the MD [29, 30]. We are convinced that materials such as braided non-absorbable sutures that create more inflammation, hence more adhesions, at the MD, are efficient and more likely to result in permanent closure than monofilaments. In a large registry-based study, Stenberg et al. concluded that using non-absorbable running sutures was probably slightly more effective than using metal clips [43]. Yang et al. also concluded, albeit only in a small study, that using running sutures was more effective that using separate stitches [44]. With experience, the time needed to close does not depend on the suture material, so that we recommend using running braided non-absorbable sutures.

In the present study, most patients requiring reoperation for IH had acute symptoms that warranted emergency surgery. Despite this, many of them could be treated using a laparoscopic approach, and conversion to open surgery was only necessary in a minority. An open approach was primarily chosen in very few patients, most of the time by general surgeons with limited experience in MD exploration after RYGB, or in patients with extensive small bowel distension. General surgeons nowadays should be acquainted with the frequently performed RYGB procedure and minimally invasive management of its complications. Help from an experienced bariatric surgeon, however, should be sought in more complex cases, because the anatomy is often difficult to understand. In the present series, only three patients (0.1%) required a small bowel resection, and none developed short-bowel syndrome because of extensive resection. This compares well with the 0.3% rate reported by Edholm et al [45]. Apart from acute severe symptoms, IH can cause acute occasional colicky abdominal pain that resolves spontaneously. When recurrent, such symptoms suggest intermittent IH and warrant elective laparoscopic exploration. When reoperating on a patient with a history of RYGB, whatever the indication for surgery, all mesenteric defects should be explored and all those found open should be closed with running non-absorbable sutures [46]. Depending on the technique used to perform RYGB, IH can develop at unusual locations, notably between the JJ and the suture line closing the mesenteric window or the anti-obstruction stich at the JJ (Paroz’ defect), and these potential sites should also be checked [31, 47].

The present study has some limitations. First, it involves comparisons between consecutive groups of patients with different durations of follow-up and increasing surgical experience. Most IH, however, develop during the first 7 post-operative years, and all patients had a minimal duration of follow-up of 8 years, with a mean of more than 11 years in the most recent group, and 15 years in the total series. It is therefore unlikely that observed rates of IH will markedly change with longer follow-up. In a large review from the New York Statewide Planning and Research Collaborative System including more than 46,000 patients operated over a period of 11 years, experience and surgical expertise have been shown to favorably impact the rate of IH during the initial three post-operative years, with a decrease from 7.3 to 4.9% [48]. Experience probably also plays a role in the present study, although its importance is impossible to evaluate. The initial group, however, includes 409 patients, so that a large experience had been developed even before the first major technical change was made. On the other side, this study has strengths in that the groups are relatively large with high follow-up rates even in the long-term, and that duration of follow-up by far exceeds the mean interval to IH, so that most IH events are captured.

## Conclusions

Implementation of a meticulous technique using running braided non-absorbable sutures for closure of all MD during RYGB effectively reduces the incidence of IH, even with retrogastric and retrocolic RYGB, and this is a very important step at completion of the procedure. Despite all efforts, however, IH cannot be eliminated, and a low risk persists. A high index of suspicion remains necessary for patients who present with acute abdominal pain with/without symptoms of bowel obstruction after RYGB, with liberal use of computerized tomography and even laparoscopic exploration if in doubt.

## Data Availability

Data used in this study are available upon request to the corresponding author.

## Declarations

All procedures performed in studies involving human participants were in accordance with the ethical standards of the institutional and/or national research committee and with the 1964 Helsinki declaration and its later amendments or comparable ethical standards.

## References

[1] Adams T, Davidson LE, Litwin SE, et al. Weight and metabolic ourcomes 12 years after gastric bypass. New Engl J Med. 2017;377:1143–55. 28930514 10.1056/NEJMoa1700459 PMC5737957

[2] Duvoisin C, Favre L, Allemann P, Fournier P, Demartines N, Suter M. Roux-en-Y gastric bypass: ten-year results in a cohort of 658 patients. Ann Surg. 2018;268:1019–25. 29194086 10.1097/SLA.0000000000002538

[3] Jiménez A, Ibarzabal A, Moizé V, et al. Ten-year outcome after Roux-en-Y gastric bypass and sleeve gastrectomy: an observational nonrandomized cohort study. Surg Obes Relat Dis. 2019;15:382–8. 30772254 10.1016/j.soard.2019.01.020

[4] Salminen P, Grönroos S, Helmiö, et al. Effect of laparoscopic sleeve gastrectomy vs Roux-en-Y gastric bypass on weight loss, comorbidities, and reflux at 10 yeasr in adult patients with obesity: the SLEEVEPASS randomized clinical trial. JAMA Surg 2022; 157: 656–66. 10.1001/jamasurg.2022.2229 PMC9218929 35731535

[5] Fry BT, Finks JS. Abdominal pain after Roux-en-Y gastric bypass: a review. JAMA Surg. 2023;158:1096–102. 37531117 10.1001/jamasurg.2023.3211

[6] Elms C, Moon RC, Vernadore S, et al. Causes of small bowel obstruction after Roux-en-Y gastric bypass: a review of 2,395 cases at a single institution. Surg Endosc. 2014;28:1624–8. 24380988 10.1007/s00464-013-3361-1

[7] Fobi MAL, Lee H, Holness R, et al. Gastric bypass operation for obesity. World J Surg. 1998;22:925–35. 9717418 10.1007/s002689900496

[8] Podnos YD, Jimenez JC, Wilson SE, et al. Complications after laparoscopic gastric bypass. Arch Surg. 2003;138:957–61. 12963651 10.1001/archsurg.138.9.957

[9] Delko T, Kraljevic M, Köstler T, et al. Primary non-closure of mesenteric defects in laparoscopic Roux-en-Y gastric bypass: reoperations and intraoperative findings in 146 patients. Surg Endosc. 2016;30:2367–73. 26335072 10.1007/s00464-015-4486-1

[10] Higa K, Ho T, Boone KB. Internal hernia after laparoscopic Roux-en-Y gastric bypass: incidence, treatment and prevention. Obes Surg. 2003;13:350–4. 12841892 10.1381/096089203765887642

[11] Paroz A, Calmes JM, Giusti V, et al. Internal hernia after laparoscopic Roux-en-Y gastric bypass for morbid obesity: a continuous challenge in bariatric surgery. Obes Surg. 2006;16:1482–7. 17132415 10.1381/096089206778870102

[12] Ianelli A, Senni Buratti M, Novellas S, et al. Internal hernia as a complication of laparoscopic Roux-en-Y gastric bypass. Obes Surg. 2007;17:1283–6. 18008110 10.1007/s11695-007-9229-5

[13] Cho M, Pinto D, Carrodegas S, et al. Frequency and management of internal hernias after laparoscopic antecolic antegastric Roux-en-Y gastric bypass without division of the small bowel mesentery or closure of mesenteric defects: review of 1400 consecutive cases. Surg Obes Relat Dis. 2006;2:87–91. 16925328 10.1016/j.soard.2005.11.004

[14] Champion K, Williams M. Internal hernia and small bowel obstruction after laparoscopic Roux-en-Y gastric bypass. Obes Surg. 2003;13:596–600. 12935361 10.1381/096089203322190808

[15] Finnel CW, Madan AK, Tichanski DS, et al. Non-closure of defects during laparoscopic Roux-en-Y gastric bypass. Obes Surg. 2007;17:145–8. 17476862 10.1007/s11695-007-9038-x

[16] Brolin RE, Kella VN. Impact of complete mesenteric closure on small bowel obstruction and internal mesenteric hernia after laparoscopic Roux-en-Y gastric bypass. Surg Obes Relat Dis. 2013;9:850–5. 23415691 10.1016/j.soard.2012.11.007

[17] Carmody B, DeMaria EJ, Jamal M, et al. Internal hernia after laparoscopic Roux-en-Y gastric bypass. Surg Obes Realt Dis. 2005;1:543–8. 10.1016/j.soard.2005.08.005 16925288

[18] De la Cruz MN, Cabrera JC, Cuesta M, et al. Closure of mesenteric defect can lead to decrease in internal hernias after Roux-en-Y gastric bypass. Surg Obes Relat Dis. 2011;7:176–80. 21126922 10.1016/j.soard.2010.10.003

[19] Miyashiro LA, Fuller WD, Ali MR. Favorable internal hernia rate achieved using retrocolic, retrogastric alimentary limb in laparoscopic Roux-en-Y gastric bypass. Surg Obes Relat Dis. 2010;6:158–64. 20359667 10.1016/j.soard.2009.12.005

[20] Obeid A, McNeal S, Breland M, et al. Internal hernia after laparoscopic Roux-en-Y gastric bypass. J Gastrointest Surg. 2014;18:250–6. 24101451 10.1007/s11605-013-2377-0

[21] Rosas U, Ahmed S, Leva N, et al. Mesenteric defect closure in laparoscopic Roux-en-Y gastric bypass: a randomized controlled trial. Surg Endosc. 2015;29:2486–90. 25480607 10.1007/s00464-014-3970-3

[22] Stenberg E, Szabo E, Ågren G, et al. Closure of mesenteric defects in laparoscopic gastric bypass: a multicentre, randomised, parallel, open-label trial. Lancet. 2016;387:1397–404. 26895675 10.1016/S0140-6736(15)01126-5

[23] Magioulotis DE, Tzovaras G, Tasiopoulou VS, et al. Closure of mesenteric defects in laparoscopic gastric bypass: a meta-analysis. Obes Surg. 2020;30:1935–43. 31955371 10.1007/s11695-020-04418-2

[24] Hajibandeh S, Hajibandeh S, Abdelkarim M, et al. Closure versus non-closure of mesenteric defects in laparoscopic Roux-en-Y gastric bypass: a systematic review and meta-analysis. Surg Endosc. 2020;34:3306–20. 32270276 10.1007/s00464-020-07544-1

[25] Muir D, Choi B, Clements C, et al. Mesenteric defect closure and the rate of internal hernia in laparoscopic Roux-en-Y gastric bypass: a systematic review and meta-analysis. Obes Surg. 2023;33:2229–36. 37162714 10.1007/s11695-023-06597-0

[26] Wu QL, Liu QZ, Xi YY, et al. Closed or unclosed mesentery? A meta-analysis of internal herniation after laparoscopic Roux-en-Y gastric bypass. Obes Surg. 2023;33:1900–9. 37081253 10.1007/s11695-023-06594-3

[27] Stenberg E, Ottosson J, Magnuson A, et al. Long-term safety and efficacy of closure of mesenteric defects in laparoscopic gastric bypass surgery: a randomized clinical trial. JAMA Surg. 2023;158:709–17. 37163240 10.1001/jamasurg.2023.1042 PMC10173104

[28] Mahmmadi-Zaniani G, Quake SYL, Musbahi A, et al. Establishing methods of defect closure in Roux-en-Y gastric bypass: an international survey. Obes Surg. 2023;33:1049–59. 36609742 10.1007/s11695-022-06420-2

[29] Skidmore A, Aarts EO. Preventing Peterson’s space hernia using a BIO synthetic mesh. BMC Surg. 2021;21:236. 33947376 10.1186/s12893-021-01197-0 PMC8097920

[30] Love LW, Mansour R, Hale AL, et al. Use of bioabsorbable tissue reinforcement reduces incidence of internal hernia in Roux-en-Y gastric bypass patients. Am Surg. 2018;84:1756–61. 30747629

[31] Paroz A, Calmes JM, Romy S, et al. A new type of intermnal hernia after laparoscopic Roux-en-Y gastric bypass. Obes Surg. 2009;19:527–30. 19034588 10.1007/s11695-008-9770-x

[32] Abasbassi M, Pottel H, Deylgat B, et al. Small bowel obstruction after antecolic antegastric laparoscopic Roux-en-Y gastric bypass without division of small bowel mesentery: a single-centre, 7-year review. Obes Surg. 2011;21:1822–7. 21656166 10.1007/s11695-011-0462-6

[33] Schneider R, Schulenburg M, Kraljevic M, et al. Does the non-absorbable suture closure of the jejunal mesenteric defect reduce the incidence and severity of internal hernias after laparoscopic Roux-en-Y gastric bypass? Langenbecks Arch Surg. 2021;406:1831–8. 34021417 10.1007/s00423-021-02180-2 PMC8481144

[34] Aghajani E, Nergaard BJ, Leifson BG, et al. The mesenteric defects in laparoscopic Roux-en-Y gastric bypass: 5 years follow-up of non-closure versus closure using the stapler technique. Surg Endosc. 2017;31:3743–8. 28205037 10.1007/s00464-017-5415-2 PMC5579176

[35] Ben Amor I, Kassir R, Debs T, et al. Impact of mesenteric defect closure during laparoscopic Roux-en-Y gastric bypass (LRYGB): a retrospective study for a total of 2093 LRYGB. Obes Surg. 2019;29:3342–7. 31175558 10.1007/s11695-019-04000-5

[36] Blockhuys M, Gypen B, Heyman S, et al. Internal hernia after laparoscopic gastric bypass: effect of closure of the Petersen defect - single-center study. Obes Surg. 2019;29:70–5. 30167987 10.1007/s11695-018-3472-9

[37] Chowbey P, Bayjal M, Khantari RS, et al. Mesenteric defect closure decreases the incidence of internal hernias following laparoscopic Roux-en-Y gastric bypass: a retrospective cohort study. Obes Surg. 2016;26:2029–34. 26757920 10.1007/s11695-016-2049-8

[38] Samur JS, Hurtado MB, Perez-Castilla A, et al. Effect of the closure of mesenteric defects in laparoscopic Roux-en-Y gastric bypass: a prospective study. Surg Obes Relat Dis. 2019;15:1903–7. 31521564 10.1016/j.soard.2019.08.005

[39] Nuytens F, D’Hondt M, Van Rooj F, et al. Closure of mesenteric defects is associated with a higher incidence of small bowel obstruction due to adhesions after laparoscopic antecolic Roux-en-Y gastric bypass: a retrospective cohort study. Int J Surg. 2019;71:149–55. 31542389 10.1016/j.ijsu.2019.09.017

[40] Stenberg E, Näslund E, Szabo E, et al. Impact of mesenteric defect closure technique on complications after gastric bypass. Langenbecks Arch Surg. 2018;403:481–6. 29858618 10.1007/s00423-018-1684-z PMC6013510

[41] Rondelli F, Bugiantella W, Desio M, et al. antecolic or retrocolic alimentary limb in laparoscopic Roux-en-Y gastric bypass? A meta-analysis Obes Surg. 2016;26:182–5. 26456394 10.1007/s11695-015-1918-x

[42] Al Harake AB, Kallies KJ, Bogert AJ, et al. Bowel obstruction rates in antecolic/antegastric versus retrocolic/retrogastric Roux limb gastric bypass: a meta-analysis. Surg Obes relat Dis. 2016;12:194–8. 26003892 10.1016/j.soard.2015.02.004

[43] Stenberg E, Ottosson J, Szabo E, et al. Comparing techniques for mesenteric defects closure in laparoscopic gastric bypass surgery—a register-based cohort study. Obes Surg. 2019;29:1129–35. 10.1007/s11695-018-03670-x 30675687

[44] Yang J, Guan B, Huang S, et al. Different surgical techniques that influenced internal hernia prevalence rate after laparoscopic Roux-en-Y gastric bypass: a retrospective analysis of 331 cases. BMC Surg. 2020;20:48. 32178649 10.1186/s12893-020-00713-y PMC7077004

[45] Edholm D, Hofgård JO, Andersson E, et al. Very low risk of short bowel after Roux-en-Y gastric bypass – a large nationwide Swedish cohort study. Surg Obes Relat Dis. 2024;20:362–6. 10.1016/j.soard.2023.10.014 38114384

[46] Zaigham H, Ekelund M, Regnér S. Long-term follow-up and risk of recurrence of internal herniation after Roux-en-Y gastric bypass. Obes Surg. 2023;33:2311–6. 37266865 10.1007/s11695-023-06653-9 PMC10344975

[47] Patel K, Serapiglia V, Rizzo AN, et al. A paradoxical internal hernia at Brolin’s anti-obstruction stitch following Roux-en-Y gastric bypass: a case report. J Surg Case Rep. 2023;10:1–2. 10.1093/jscr/rjad554 PMC10587004 37867920

[48] Ende V, Devas N, Zhang X, et al. Internal hernia trends following gastric bypass surgery. Surg Endosc. 2023;37:7183–91. 37349593 10.1007/s00464-023-10206-7

